# A patient with hypereosinophilic syndrome that manifested with acquired hemophilia and elevated IgG4: a case report

**DOI:** 10.1186/1752-1947-6-63

**Published:** 2012-02-14

**Authors:** Yoshiro Nagao, Hiromi Yamanaka, Hiromasa Harada

**Affiliations:** 1 Department of Internal Medicine, Yao Tokushukai General Hospital, 1-11 Wakakusa-cho, Yao city, Osaka, 581-0011, Japan; 2 Department of Pathology, Yao Tokushukai General Hospital, 1-11 Wakakusa-cho, Yao city, Osaka, 581-0011, Japan

## Abstract

**Introduction:**

Hypereosinophilic syndrome is defined as a prolonged state (more than six months) of eosinophilia (greater than 1500 cells/μL), without an apparent etiology and with end-organ damage. Hypereosinophilic syndrome can cause coagulation abnormalities. Among hypereosinophilic syndrome types, the lymphocytic variant (lymphocytic hypereosinophilic syndrome) is derived from a monoclonal proliferation of T lymphocytes. Here, we describe the case of a patient with lymphocytic hypereosinophilic syndrome who presented with a coagulation abnormality. To the best of our knowledge, this is the first such report including a detailed clinical picture and temporal cytokine profile.

**Case presentation:**

A 77-year-old Japanese man presented to our facility with massive hematuria and hypereosinophilia (greater than 2600 cells/μl). His eosinophilia first appeared five years earlier when he developed femoral artery occlusion. He manifested with multiple hematomas and prolonged activated partial thromboplastin time. His IgG4 level was remarkably elevated (greater than 2000 mg/dL). Polymerase chain reaction tests of peripheral blood and bone marrow identified lymphocytic hypereosinophilic syndrome. His prolonged activated partial thromboplastin time was found to be due to acquired hemophilia. Glucocorticoids suppressed both the hypereosinophilia and coagulation abnormality. However, tapering of glucocorticoids led to a relapse of the coagulation abnormality alone, without eosinophilia. Tumor necrosis factor α, interleukin-5, and/or eotaxin-3 may have caused the hypereosinophilia, and interleukin-10 was correlated with the coagulation abnormality.

**Conclusions:**

To the best of our knowledge, this is the first case in which lymphocytic hypereosinophilic syndrome and IgG4-related disease have overlapped. In addition, our patient is only the second case of hypereosinophilic disease that manifested with acquired hemophilia. Our patient relapsed with the coagulation abnormality alone, without eosinophilia. This report shows that the link between eosinophilia, IgG4, and clinical manifestations is not simple and provides useful insight into the immunopathology of hypereosinophilic syndrome and IgG4-related disease.

## Introduction

Hypereosinophilic syndrome (HES) was originally proposed as a state of (i) blood eosinophilia with an absolute eosinophil count greater than 1500 cells/μL and persisting for more than six months, (ii) without an apparent etiology (for example, parasitic infection or allergic disease), and (iii) with eosinophil-mediated organ dysfunction [1]. Currently, HES is classified based on etiology [2]. For example, the lymphocytic variant (L-HES) is derived from a monoclonal proliferation of T lymphocytes. T cell clones identified in L-HES often express aberrant immunophenotypes (for example, CD3-CD4+, CD3+CD4-CD8-, CD4+CD7-, CD16+CD56+) [3-6], although no aberrancy has been identified in many patients with L-HES [7]. In contrast, the myeloproliferative variant (M-HES) is characterized by emergence of fusion genes (for example, *PDGFRa*, *PDGFRb*, and *FGFR1*) originating from a chromosomal translocation in 4q12, 5q33, and 8p11, respectively [8]. HES affects not only diverse organs, but also causes thrombotic occlusion in arteries [9,10], veins [11-14], and capillaries [15]. In addition, patients with HES present with coagulation abnormalities, especially disseminated intravascular coagulation (DIC), possibly due to this thrombotic tendency [9,14,16-18]. However, the mechanism of HES-derived coagulation abnormality is not fully understood.

## Case presentation

**Figure 1:**
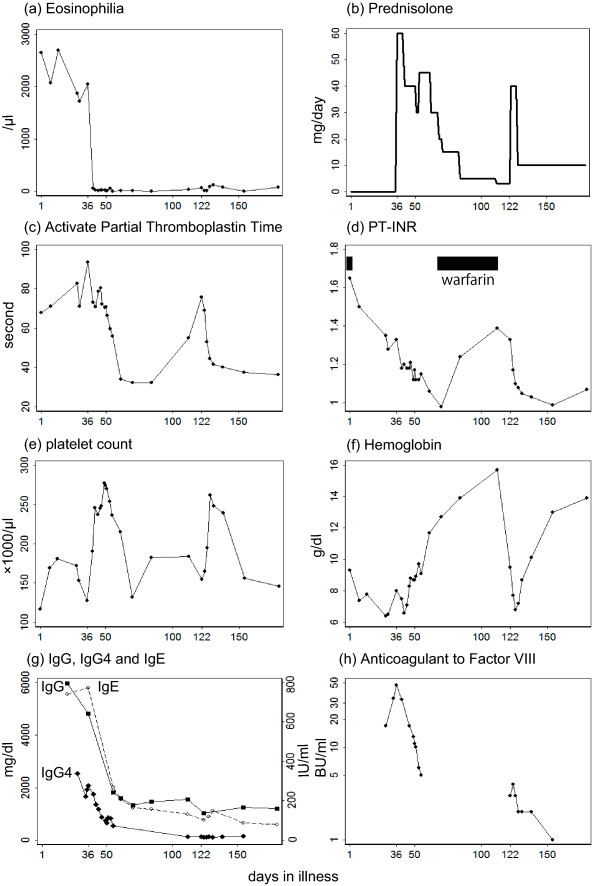
**Temporal profile of the biological manifestations and treatment of our patient**. **(a) **Eosinophil count, **(b) **prednisolone (intravenous and oral), **(c) **activated partial thromboplastin time, **(d) **prothrombin time-international normalized ratio (PT-INR), **(e) **platelet count, **(f) **hemoglobin level, **(g) **IgG, IgG4 (mg/dL) and IgE (IU/mL), and **(h) **anticoagulant to factor VIII, are plotted above the time axis.

A 77-year-old Japanese man presented to the out-patient department of our hospital (day one) with hematuria (42,000 red blood cells/μL) and a hematoma under the jaw. He had anemia (hemoglobin (Hb) 9.3 g/dL), and a remarkable eosinophilia (2600 cells/μL; 32% of white blood cells) (Figure 1a). He had a history of minor bronchial asthma, which had been treated with an inhaled glucocorticoid and an anticholinergic. His medical records also showed a history of hypereosinophilia five years earlier, when he developed femoral artery occlusion. He was prescribed warfarin 2 mg/day after the associated bypass surgery. In spite of this history, vascular risk factors (diabetes mellitus, hypertension, and hyperlipidemia) were absent. At presentation, renal damage (β2-macroglobulin 9.5 mg/L, normal range 0.9 to 1.9 mg/L) was recognized. High levels of IgG and IgE (Figure 1g) were also noted. IgA (90 mg/dL) and IgM (36 mg/dL) were within normal ranges. While his platelet count (169,000 cells/μL) and bleeding time (one and a half minutes, normal range one to three minutes) were normal, coagulation times were remarkably prolonged: the activated partial thromboplastin time (APTT) was 68 seconds (normal: 25 to 40 seconds) and the prothrombin time-international normalized ratio (PT-INR) was 1.65 (Figure 1c,d). Since hematuria and hematoma were assumed to be due to this coagulopathy, warfarin was stopped.

**Figure 2:**
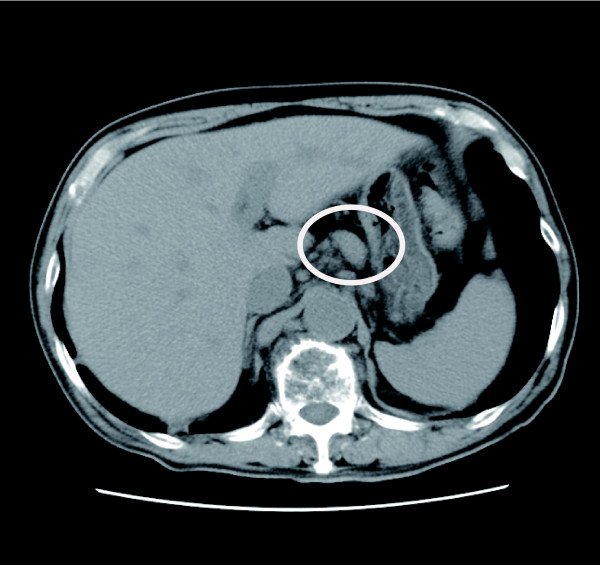
**Enlarged lymph nodes near the abdominal aorta at the onset of illness**. The enlarged lymph nodes are indicated.

We considered bronchial asthma, eosinophilic pneumonia, Churg-Strauss syndrome (CSS), malignancies [19], parasitic infections, adrenal insufficiency, and HES as differential diagnoses to explain this eosinophilia. However, his minor bronchial asthma did not explain the high level of eosinophilia. Since a computed tomography (CT) scan detected no abnormalities in the lungs, eosinophilic pneumonia was excluded. Among six diagnostic criteria for CSS, only asthma and eosinophilia were fulfilled [20]. In addition, tests for anti-neutrophil cytoplasmic antibodies (ANCAs) were negative. Therefore, CSS was unlikely. Tumor markers were measured (carcinoembryonic antigen (CEA), squamous cell carcinoma (SCC), α-fetoprotein (AFP), and protein induced by vitamin K absence 2 (PIVKA2)) and M-protein findings were all negative. There were no parasitic eggs or larvae in his stool, and results of sera anti-*Aspergillus *antibody tests were negative. His cortisol level was within the normal range on repeated measurements; hence, adrenal insufficiency was considered unlikely. Based on these results, HES remained as the most likely diagnosis. To categorize the HES, monoclonality in the T cell receptor (TCR) was examined by polymerase chain reaction (PCR) [21] (Mitsubishi Medience, Tokyo, Japan), using peripheral blood and bone marrow aspirate. Subsequently, in both samples, TCR monoclonality was detected in β, γ, and δ chains (Table 1). This finding supported the diagnosis of L-HES. Consistent with this diagnosis, multiple enlarged lymph nodes near the abdominal aorta were detected on CT scan (Figure 2). In contrast, no chromosome abnormality was detected in the bone marrow aspirates. In addition, fluorescence *in situ *hybridization (FISH) analysis of peripheral blood and bone marrow aspirates did not detect 4q12 translocation, the most frequent translocation associated with M-HES. Therefore, M-HES was less likely [2]. Collectively, these findings pointed to a diagnosis of L-HES.

**Table 1:** Monoclonalities detected by polymerase chain reaction (PCR) in the T-cell receptor genes

Chain	Region to which PCR was applied	Bone marrow	Peripheral blood
β Chain	Vβ/Jβ1,2	Positive	Positive
	Vβ/Jβ2	Positive	Positive
	Dβ/Jβ	Positive	Positive
γ Chain	Vγ If, Vγ10/Jγ	Positive	Positive
	Vγ 9, Vγ11/Jγ	Positive	Positive
δ Chain	Vδ/Jδ	Positive	Marginal

Positive: monoclonality was detected. Marginal: the peak of PCR was noticeable but its height was lower than the positive control. The methodology is described in detail in [21].

After warfarin was stopped from day one, his PT-INR steadily normalized (Figure 1d). However, APTT continued to be prolonged (Figure 1c). Although our patient tested positive for anti-hepatitis B core (HBc) and anti-hepatitis C virus (HCV) antibodies, hepatitis B surface (HBs) antigen, hepatitis B virus (HBV) DNA and HCV RNA were all undetectable. Transaminases were consistently within the normal range. Ultrasonography, CT, and MRI scans did not detect any abnormality in the liver. Hence, the liver was not the source of the prolonged APTT. Other causes of a coagulation abnormality (such as protein C and/or S deficiencies, systemic lupus erythematosus, and anti-phospholipid antibody syndrome) were excluded (data not shown). Although the possibility of DIC was suggested (fibrin degradation products (FDP) 18 μg/mL, D-dimer 7.4 μg/mL, anti-thrombin III 66%, platelet count 117,000 cells/μL), ultrasonography detected no thrombus in the heart or veins/arteries of the lower limbs. Taken together, the findings suggested the prolonged APTT in our patient most likely originated from his HES.

On day 28, our patient developed a subcutaneous hematoma in his hip, which spread rapidly to the thighs, and he was admitted to our hospital. His APTT was further prolonged (83 seconds; Figure 1c). Hematomas appeared on his trunk and upper limbs. His oral mucosa and a scar in his ear began to bleed. Although a large amount of fresh frozen plasma and red blood cells were infused between days 31 and 34, his APTT reached a high of 93 seconds on day 36, and the hemoglobin level decreased to 7.9 g/dL (Figure 1c,f). On day 36, prednisolone was started at 60 mg/day (Figure 1b). His peripheral eosinophils decreased quickly (Figure 1a). The APTT and platelet count gradually normalized (Figure 1c,e), while bleeding, subcutaneous hematomas, hematuria, and anemia subsided steadily (Figure 1f). Renal damage improved (β2-macroglobulin decreased to 3.4 mg/L). A CT scan revealed that the lymph nodes around the abdominal aorta had shrunk to a normal size (data not shown). His γ-globulinemia normalized (Figure 1g). He was discharged on day 55. Prednisolone was continuously tapered in the out-patient setting (Figure 1b).

On day 122, when the dose of prednisolone was down to 3 mg/day, a subcutaneous hematoma developed in his thigh. Although his eosinophil count was normal at 64 cells/μL (0.4% of the total white blood cells), his APTT was again prolonged (76 seconds, Figure 1c). He was admitted to our hospital again, where prednisolone was raised to 40 mg/day (Figure 1b). Since his APTT then normalized and hematoma diminished steadily, he was discharged on day 141. To date, he has been kept on prednisolone at 10 mg/day, with no further relapses of coagulopathy or peripheral hypereosinophilia.

**Figure 3:**
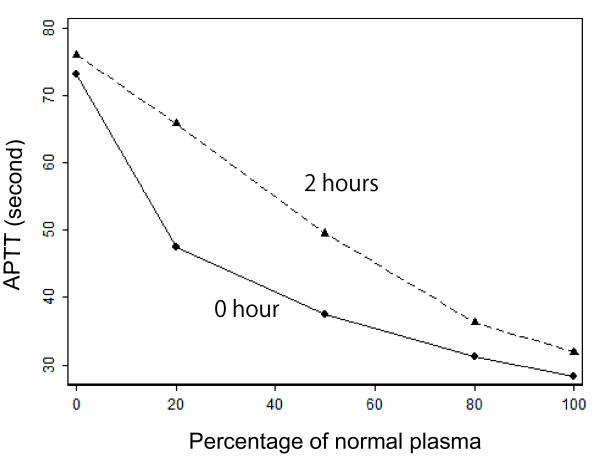
**Mixing test**. The activated partial thromboplastin time (APTT) was measured on day 122 with our patient's plasma mixed with normal plasma collected from healthy individuals, in various proportions. APTT was measured immediately after the mixing (solid line) and after a two-hour incubation at 37°C (dashed line). The incubation prolonged APTT as compared to no incubation, suggesting the presence of a circulating anticoagulant.

To investigate the prolonged APTT, coagulation factors V to XII were measured. Consequently, the activity of factor VIII was markedly reduced: 4% on day 122 (the day of relapse onset). Other factors were all within normal ranges (data not shown). To examine the pathogenesis of the coagulopathy, we preserved our patient's sera (at -80°C) taken after day 34. Control sera were also obtained from 10 healthy individuals (ages 29 to 59 years old, five men, five women). Written informed consent was obtained from all individuals. The research protocol was approved by the committee for clinical ethics of Yao Tokushukai General Hospital. Circulating anticoagulant against factor VIII was evaluated in the preserved sera by using the Bethesda assay. As a result, our patient's anticoagulant level was found to be elevated during both the initial and relapse episodes: 47 BU/mL on day 36, 4 BU/mL on day 124 (Figure 1h), while anticoagulant was not detected in any of the control sera. Furthermore, the result of a cross-mixing test conducted on day 122 was not inconsistent with acquired hemophilia (that is, acquisition of anticoagulant) (Figure 3). Our patient and his parents had no history of bleeding abnormalities, which excluded hereditary hemophilia. Therefore, acquired hemophilia was the most likely diagnosis. Other than anticoagulant against factor VIII, all the autoantibodies examined (including anti-nuclear, anti- Sjögren's syndrome A/B (SS-A/B), anti-thyroglobulin, anti-thyroid peroxidase, and anti-DNA antibodies) showed normal values.

**Figure 4:**
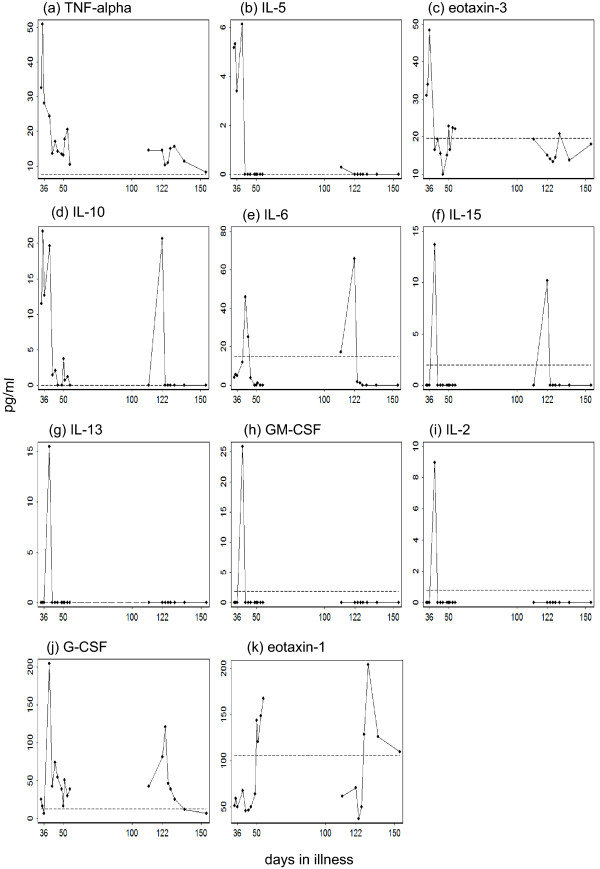
**Temporal profile of cytokines in our patient's sera**. The following 15 cytokines were measured in the sera taken after day 34: interleukin (IL)-2, IL-3, IL-4, IL-5, IL-6, IL-10, IL-13, IL-15, IL-17, eotaxin-1, eotaxin-3, granulocyte colony stimulating factor (G-CSF), granulocyte-macrophage colony stimulating factor (GM-CSF), tumor necrosis factor α (TNFα), and interferon γ (IFNγ). The dashed line in the each panel represents the upper limit of the 95% confidence interval (95% CI) estimated from the healthy controls. Day 36 (when prednisolone was started at 60 mg/day) and day 122 (when prednisolone was raised from 3 mg/day to 40 mg/day) are specifically labeled on the time axis. IL-3, IL-4, IL-17, and IFNγ, which were consistently within the 95% CI of controls, were thus omitted.

To illustrate the immunological dynamics in our patient, 15 cytokines in the sera were measured by using a Milliplex assay (Millipore, Billerica, MA, USA) (Figure 4). To examine the possible involvement of IgG4-related disease (IgG4RD), a newly recognized disease entity [22], we measured IgG4 in the preserved serum. IgG4 showed a disproportionately high value (Figure 1g). For example, on day 36, IgG4 was 2070 mg/dL (normal range 4.8 to 105 mg/dL), which constituted 48% of the total IgG (normal value < 7%).

## Discussion

We report a case of a patient with L-HES who manifested with a coagulation abnormality. Glucocorticoids effectively suppressed hypereosinophilia and corrected the coagulation abnormality. However, excessive subsequent tapering of the glucocorticoid brought about a relapse of the coagulation abnormality, but not the eosinophilia.

It has been reported that HES can manifest with thrombotic tendency. Our patient had developed femoral artery occlusion, a frequent manifestation of HES-induced thrombosis, five years prior to the episode reported here. The thrombotic tendency had been controlled effectively by warfarin. In the episode reported here, factor VIII activity was remarkably decreased, which was most likely due to acquired hemophilia. Hypereosinophilia complicated with acquired hemophilia has been reported only once previously in the literature [23]. In our patient, DIC, a frequent complication of HES [9,14,16-18], possibly exacerbated the bleeding tendency. These findings remain to be generalized to other cases of HES-derived coagulopathy.

Our patient's case, featuring an extremely high value of IgG4, is likely to be an example of IgG4RD. IgG4RD, first reported in 1993 [24], is characterized by elevated serum IgG4 and/or tissue infiltration by IgG4+ plasma cells [25,26]. IgG4RD has been recognized as a systemic illness which affects numerous organs including lymph nodes [24], pancreas [25], salivary gland [24,26], retroperitoneum [27], and kidney [28]. Its pathogenesis, however, remains to be elucidated [22]. IgG4RD has been often associated with eosinophilia [29-31], and responds well to glucocorticoid [22]. These characteristics are consistent with our patient, supporting the diagnosis of IgG4RD in this case. To the best of our knowledge, the presented case is the first reported example in which L-HES and IgG4RD have overlapped.

The cytokines measured in our patient's sera can be classified into three groups in relation to the illness (Figure 4). First, tumor necrosis factor α (TNFα), interleukin (IL)-5, and eotaxin-3 increased to very high levels immediately before the first episode, and decreased to low levels after the glucocorticoid was started on day 36. Therefore, these cytokines were correlated with eosinophilia. Second, IL-10 was present at very high levels until glucocorticoid was started on day 36, and peaked again at the onset of relapse. Hence, IL-10 exhibited a strong correlation with prolonged APTT and anticoagulant to factor VIII. Third, other cytokines in Figure 3 rose to high levels immediately after day 36. These peaks may possibly be reactions to substances released from dying eosinophils [9,18]. Among the cytokines in this group, granulocyte colony stimulating factor (G-CSF) and eotaxin-1 showed second elevations in the relapse phase, suggesting heterogeneity within this group. The roles of these cytokines in the pathogenesis are difficult to explain. Collectively, TNFα, IL-5, and eotaxin-3 appeared to play an important role in generating peripheral hypereosinophilia, while IL-10 was most closely correlated with prolonged APTT and anticoagulant. Indeed, eosinophilic diseases have been associated with TNFα [32,33], IL-5 [34], IL-10 [35], and eotaxin-3 [36,37], while IL-10 was overexpressed in IgG4RD [38,39]. The causal relationship between these cytokines, eosinophilia, IgG4, and clinical manifestations remains to be elucidated.

## Conclusions

To the best of our knowledge, this is the first case of L-HES overlapped with IgG4RD, and the second reported case of hypereosinophilic disease complicated by acquired hemophilia. Furthermore, this is the first case of L-HES or IgG4RD in which the cytokine profile was described during the phases of onset and relapse. Although the clinical manifestation (that is, coagulopathy) presented twice (in the first episode and in the relapse), the underlying immunological profiles were dissimilar between these two periods. These findings imply that the interaction between HES and IgG4RD is a complex process.

## Consent

Written informed consent was obtained from the patient for publication of this case report and any accompanying images. A copy of the written consent is available for review by the Editor-in-Chief of this journal.

## Competing interests

The authors declare that they have no competing interests.

## Authors' contributions

YN analyzed and interpreted the data from our patient regarding the hypereosinophilic syndrome. HY performed all of the examinations, including hematological and immunological, and took responsibility for data management. HH took responsibility for the clinical management of our patient. All authors read and approved the final manuscript.
